# Comparative susceptibility of children and adults to neurological effects of inhaled manganese: A review of the published literature^☆^

**DOI:** 10.1016/j.envres.2023.115319

**Published:** 2023-01-17

**Authors:** Rachel M. Shaffer, J. Michael Wright, Ila Cote, Thomas F. Bateson

**Affiliations:** a Center for Public Health and Environmental Assessment, Office of Research and Development, U.S. Environmental Protection Agency, Washington, DC, USA; b Center for Public Health and Environmental Assessment, Office of Research and Development, U.S. Environmental Protection Agency, Cincinnati, OH, USA; c University of Colorado, School of Public Health, Aurora, CO, USA

**Keywords:** Manganese, Neurotoxicity, Motor effects, Cognitive effects, Lifestages

## Abstract

**Background:**

Manganese (Mn) is neurotoxic in adults and children. Current assessments are based on the more extensive adult epidemiological data, but the potential for greater childhood susceptibility remains a concern. To better understand potential lifestage-based variations, we compared susceptibilities to neurotoxicity in children and adults using Mn biomarker data.

**Methods:**

We developed a literature search strategy based on a Population, Exposures, Comparators, and Outcomes statement focusing on inhalation exposures and neurological outcomes in humans. Screening was performed using DistillerSR. Hair biomarker studies were selected for evaluation because studies with air measurements were unavailable or considered inadequate for children. Studies were paired based on concordant Mn source, biomarker, and outcome. Comparisons were made based on reported dose-response slopes (children vs. adults). Study evaluation was conducted to understand the confidence in our comparisons.

**Results:**

We identified five studies evaluating seven pairings of hair Mn and neurological outcomes (cognition and motor effects) in children and adults matched on sources of environmental Mn inhalation exposure. Two Brazilian studies of children and one of adults reported intelligent quotient (IQ) effects; effects in both comparisons were stronger in children (1.21 to 2.03-fold difference). In paired analyses of children and adults from the United States, children exhibited both stronger and weaker effects compared to adults (0.37 to 1.75-fold differences) on postural sway metrics.

**Conclusion:**

There is limited information on the comparative susceptibility of children and adults to inhaled Mn. We report that children may be 0.37 to 2.03 times as susceptible as adults to neurotoxic effects of Mn, thereby providing a quantitative estimate for some aspects of lifestage variation. Due to the limited number of paired studies available in the literature, this quantitative estimate should be interpreted with caution. Our analyses do not account for other sources of inter-individual variation. Additional studies of Mn-exposed children with direct air concentration measurements would improve the evidence base.

## 1. Introduction

Manganese (Mn), a naturally occurring element, is an essential nutrient required for proper enzyme functioning and cellular respiration across multiple organ systems, including the brain (Institute of Medicine (US) Panel on Institute of Medicine Panel on Micronutrients, 2001; Wedler 1993). However, at elevated concentrations, Mn is a neurotoxicant linked to adverse cognitive and motor outcomes in both children and adults (ATSDR 2012; Liu et al., 2020; Lucchini et al., 2018; U.S. Environmental Protection Agency (EPA) 1993; WHO 2001). Exposure to excess Mn in the general population may occur via either inhalation or oral routes, including through food, water, and air pollutants. Infants are primarily exposed to Mn via breastfeeding or use of baby formula (Lucchini et al., 2017; Scher et al., 2021). Diet is also the main source of exposure to excess Mn for the general population (ATSDR, 2012), but normally only 3–5% of ingested Mn is retained due to rapid elimination from the blood through first-pass metabolism in the liver and biliary excretion (Davidsson et al. 1988, 1989; Schwartz et al., 1986). Inhaled Mn partially avoids the homeostatic regulations (i.e., first-pass metabolism) that control internal Mn concentrations as inhaled Mn enters through the pulmonary circulation rather than the hepatic circulation (Aschner et al., 2005). A particular concern is that, compared to ingested Mn, inhaled Mn is more likely to enter the brain because 15% of blood flow will enter the central nervous system after exiting the pulmonary circulation without being processed in the liver (Andersen et al., 1999; Aschner and Dorman, 2006; Xing et al., 2017). Non-circulatory pathways exist for ambient Mn as well. Rodent and non-human primate studies demonstrate the potential for particle size-dependent transfer from the nose to the brain through the olfactory nerve (Brenneman et al., 2000; Dorman et al. 2006a, 2006b; Elder et al., 2006; Fechter et al., 2002); passage through the trigeminal nerve has also been observed (Aschner and Dorman, 2006; Lewis et al., 2005). Data indicate that inhaled Mn accumulates in the iron-rich basal ganglia region of the brain (Nelson et al., 1993). The basal ganglia plays an essential role in motor control as well as sensory processing and cognition (Brown et al., 1997; Visser and Bloem, 2005).

In the United States (U.S.), Mn is emitted by iron and steel production plants, ferroalloy producers, power plants, coke ovens, many smaller metal processing facilities, and vehicles. Reporting from 37 air monitoring sites in the midwestern U.S., the median average annual ambient concentrations of Mn ranged from 0.012 μg/m^3^ to 0.063 μg/m^3^ (U. S. Environmental Protection Agency (EPA) 2018). Fossil fuel emissions (e.g., from naturally occurring Mn in these fuels) are the major background source. Methylcyclopentadienyl manganese tricarbonyl (MMT), a manganese compound added to enhance the octane rating of gasoline of some limited supplies, may also contribute to some ambient concentrations of Mn (ATSDR, 2012). Concentrations around industrial facilities are higher than average ambient concentrations. Ferroalloy production facilities’ maximum annual average ambient Mn concentrations are estimated to be ~1.1 μg/m^3^ (U.S. EPA, 2015). Toxic Release Inventory data for the U.S. indicate that reported on-site air releases of Mn and Mn-compounds in 2019 were approximately 439.8 and 716.9 thousand pounds, respectively (U. S. Environmental Protection Agency (EPA) 2021). There is ongoing research in the U.S. to examine exposure patterns and health outcomes in some of these industrially impacted communities (Fulk et al., 2017; Haynes et al. 2015, 2018; Martin et al., 2021; Stolfi et al., 2021), especially with regard to periodic reconsideration of ferromanganese national emissions standards as mandated by the Clean Air Act (U. S. Environmental Protection Agency (EPA) 2022).

Risks posed by airborne manganese exposure have been estimated based on neurological effects in adults exposed occupationally to airborne Mn (ATSDR 2012; Health Canada 2010; USEPA, 1993; WHO 2001). Neurologic effects associated with air manganese exposures have also been reported in children, but risk estimates based on these studies have not been developed. Most of the existing data in children are based on biomarkers, such as Mn in hair or blood. Existing physiologically-based pharmacokinetic (PBPK) models do not include hair; therefore, estimating inhaled air concentrations from hair biomarker data in children to facilitate the development of a child-specific risk estimate from inhalation exposure is not possible at this time.

It is well known that development impacts neurologic functions and consideration of inter-individual variability is important in understanding potential child and adult differences in responses (Bodmer et al., 2018). Some prior studies have evaluated concordance between available data and certain assumptions about human variability (Bokkers and Slob, 2007; Pieters et al., 1998; Price et al., 2008), but to our knowledge, there is no evaluation of this issue as it applies to Mn-induced neurotoxicity in children and adults by any route of exposure. For this publication, we conducted a paired dose-response analysis using data from the available literature to compare the susceptibility of children and adults to inhaled Mn based on hair biomarker data. We focused on hair biomarker data because health studies in children using direct air concentration measurements were unavailable.

## 2. Methods

In this section we describe: the Population, Exposures, Comparators, and Outcomes (PECO) criteria, literature search, screening of studies for further analyses pairing of child and adult studies, selection of biomarker studies, study evaluation, and comparative dose-response analyses. These methods follow standard EPA procedures.

### 2.1. Population, exposures, comparators, and outcomes (PECO) criteria

Systematic review of the scientific literature has become increasingly important in recent years for clinical evaluations, research and risk assessment (National Academies of Sciences Engineering and Medicine (NASEM) 2022). To guide our systematic review of the literature, we developed a PECO statement (Table 1) describing our focus on inhalation exposure to point sources of Mn and neurological outcomes at different lifestages. To increase the sensitivity of our analysis to detect a true association regarding potential differential susceptibility to inhaled Mn, we focused on populations near point sources of airborne Mn to better isolate situations where inhalation exposures from those sources would dominate over both nonpoint-source exposures (i.e., from fossil fuel combustion) and other pathways. Studies that did not evaluate populations with clear point-source inhalation exposures to Mn were considered supplemental, since other potential pathways of exposure would increase the noise in our analyses. Correspondingly, ambient Mn exposures among the general population resulting from nonpoint-source exposures (i.e., from fossil fuel combustion) were not the focus of this analysis.

### 2.2. Literature search, screening, and pairing

Utilizing the EPA Health and Environmental Research Online (HERO) database, we initiated a literature search for the term “manganese” in any studies published between January 1, 2011, and April 11, 2022. The literature search start date was selected to reflect publications since the most recent previous systematic review in 2012 by the U.S. Agency for Toxic Substances and Disease Registry Mn (ATSDR, 2012). Next, we used the Sciome (Howard et al., 2016) search strategy filters for “human” and (“neurological” or “musculoskeletal”). Filters are available online at https://www.sciome.com/swift-review/searchstrategies/). From here, two reviewers per study conducted Level 1 (Title and Abstract (TIAB)) screening in DistillerSR (https://www.evidencepartners.com/products/distillersr-systematic-review-software/). Conflicts were resolved through conversations between reviewers, with consultation by a third reviewer if needed. Next, a Level 2 (Full-Text) screen was conducted by two reviewers to confirm relevance to the PECO statement, with conflicts resolved through discussion.

Additional studies were identified through citation mapping from relevant recent systematic reviews. References from seed articles were included in the literature database, cross-referencing with existing records and removing duplicates. These studies were imported directly into DistillerSR for PECO eligibility screening through TIAB and potential full-text review.

Data on population, exposure source, exposure biomarker, outcome, and outcome evaluation approach were extracted from the selected studies. Paired studies were identified based on the concordance of Mn source, biomarker, and outcome. Studies conducted in similar or overlapping study populations were prioritized for pairing.

### 2.3. Biomarker selection

There is currently no gold-standard biomarker that reflects cumulative, long-term exposure to Mn (Baker et al., 2015); the optimal biomarker may depend on the route of exposure (Butler et al., 2019). Blood and urine, frequently used as biomarkers for other environmental exposures, are not suitable choices for assessing chronic inhaled Mn exposure for a variety of reasons, including that both of these biomarkers may be more influenced by dietary exposures than environmental exposures given that manganese is an essential nutrient (ATSDR, 2012); blood Mn exhibits minimal variability over a wide range of air concentrations (Smith et al., 2007), and urine accounts for only a small fraction of total Mn excretion from the body (Aschner and Aschner, 2005). Additionally, blood Mn is subject to homeostatic controls (Lucchini et al., 2012), which can complicate the interpretation of this biomarker in epidemiology and risk assessment.

Hair and nail biomarkers can reflect environmental and occupational Mn exposures (Eastman et al., 2013; Laohaudomchok et al., 2011; Liu et al., 2020), and there is increasing use of these biomarkers for assessing longer-term average Mn exposure in epidemiological studies. In particular, hair has been reported to be a reliable indicator of exposure in both children and adults (Liu et al., 2020; Reiss et al., 2016) and is less influenced by short-term variability in airborne Mn levels (Reiss et al., 2016). Additionally, hair Mn biomarkers appear to be consistently associated with neurodevelopmental outcomes (Coetzee et al., 2016; Liu et al., 2020); in limited studies assessing both hair and blood biomarkers, hair concentrations but not blood concentrations appear to be associated with neurodevelopmental outcomes in children (Menezes-Filho et al., 2011; Riojas-Rodríguez et al., 2010; Torres-Agustín et al., 2013). In contrast, nail biomarkers have not been found to be consistently associated with neurodevelopmental outcomes (Liu et al., 2020).

All biomarkers were initially considered while identifying potential pairings, but only hair biomarker studies were moved forward for study evaluation and comparative analysis. This decision was made based on the relative advantages (as noted above) of hair Mn compared to other Mn biomarkers that became apparent as we reviewed the literature (and the lack of adequate studies with direct air measurements in children). To conduct our analysis, we relied on the following interrelated simplifying assumptions: 1) the individuals had lived in the same geographic area for the duration of their lives before study participation; 2) the sources of airborne Mn remained constant over the populations’ lifetime; 3) the observed hair Mn represents chronic exposure concentrations, and 4) the observed hair Mn concentration is reflective of an earlier critical window of vulnerability for neurodevelopmental outcomes.

### 2.4. Study evaluation

A tailored study evaluation protocol was developed from prior EPA Integrated Risk Information System (IRIS) program protocols by two authors (RMS, JMW) and reviewed by subject matter experts. The general approach was consistent with established practices in the EPA IRIS program (U.S. Environmental Protection Agency (EPA) 2022) and similar to methods described previously (Keshava et al., 2020). Briefly, we assigned ratings (good, adequate, deficient, critically deficient) to individual domains (participant selection, exposure assessment, outcome ascertainment, confounding, analysis, selective reporting, sensitivity), which then contributed to an overall study evaluation rating (*high, medium, low, uninformative*).

Study evaluation on selected paired studies was conducted using the Health Assessment Workspace Collaborative (HAWC, https://hawcprd.epa.gov/portal/) by two study authors (RMS, JMW), with disputes resolved by a third author if needed (TFB).

### 2.5. Comparative dose-response analyses

Comparisons of studies using hair Mn biomarkers were made based on the reported linear slopes of the observed dose-response for the paired adult and child study populations (i.e., the beta coefficient in children compared to the beta coefficient in adults). All studies included both males and females. Sex-specific beta values were not available from the original publications, so a sex-specific comparison was not able to be examined here. Beta coefficients were assumed to be normally distributed, but within each set, the two betas may have different variances. Betas were compared using Welch’s unpaired t-tests using GraphPad (https://www.graphpad.com/quickcalcs/ttest1/?format=SEM). Differences in betas with 95% confidence intervals and p-values were obtained.

## 3. Results

### 3.1. Search results

The initial HERO search yielded 75,340 studies. Application of SWIFT review filters as described above narrowed the database to 3,442 studies. Supplemental backward searches using 7 seed studies yielded 365 additional studies. From both of these search processes, 190 studies passed from TIAB screening to full text review. A full text review identified 145 studies meeting PECO eligibility to be advanced for potential pairing. After further review for concordance of Mn biomarker and outcome, we identified thirteen studies of cognitive outcomes and eleven studies of motor outcomes (Supplemental Table S1).

### 3.2. Identification of paired studies

After filtering for studies utilizing hair biomarkers and prioritizing studies with similar point-sources of Mn, we identified pairings of three studies of cognitive outcomes and two studies of motor outcomes. No other studies reported results that used the same metrics of exposure and outcomes. Table 2 describes key population characteristics for each study.

### 3.3. Studies of IQ from Bahia, Brazil

Carvalho et al. (2014), Menezes-Filho et al. (2011), and de Sousa Viana et al. (2014) all reported on the cognitive effects of log_10_-transformed concentrations of Mn in hair among different populations in Cotegipe Village and Santa Luzia exposed from the same local ferromanganese alloy plant in operation since 1970. Carvalho et al. (2014) evaluated children ages 7–12 years. Menezes-Filho et al. (2011) evaluated children ages 6–11 years, while de Sousa Viana et al. (2014) examined adults ages 15–55 years. de Sousa Viana et al. (2014) and Carvalho et al. (2014) both measured estimated IQ with the Wechsler Intelligence Scale for Children (WISC) and the Wechsler Adult Intelligence Scale (WAIS), respectively. Menezes-Filho et al. (2011), measured full-scale IQ with the WISC.

Based on ultrafine particles that were continuously sampled in ambient air across 7 days in 2007, Menezes-Filho et al. (2009) report a mean airborne Mn concentration of 0.151 μg/m^3^ and a median concentration of 0.114 μg/m^3^, with a range of 0.011–0.439 μg/m^3^. Hair Mn concentrations showed some variability across the three populations evaluated in subsequent epidemiologic analyses: Menezes-Filho et al. (2011): mean (range) = 5.83 (0.1–86.68) μg/g for children in Cotegipe Village; Carvalho et al. (2014): median (range) = 11.48 (0.52–55.74) μg/g for children in Cotegipe Village and neighboring Santa Luzia; and de Sousa Viana et al. (2014): median (range) = 8.95 (0.6–44.6) μg/g for adults in Cotegipe Village and Santa Luzia (Table 2). Children and adults from the same two villages during the same study period showed fairly comparable median values (11.48 μg/g in children vs. 8.95 μg/g in adults) (Carvalho et al. 2014, Sousa Viana et al., 2014).

### 3.4. Studies of motor abilities from Ohio, United States

Rugless et al. (2014) and Standridge et al. (2008) reported on the association between natural log-transformed hair Mn concentrations and postural sway among different populations exposed from the same local ferromanganese smelter near Marietta, Ohio. Rugless et al. (2014) examined children ages 7–9 years, while Standridge et al. (2008) studied adults ages 31–68 years. Both studies used the same measures of postural balance (sway length and sway area) evaluated with a Hall-Effect type portable force platform system (Rugless et al., 2014; Standridge et al., 2008).

The median air Mn concentration for both studies, based on U.S. EPA Gaussian dispersion modeling software AERMOD and associated preprocessors (AERMAP, AETMET, and AERSURFACE), was estimated at 0.008 μg/m^3^ with a range of 0.001–0.054 μg/m^3^ (Haynes et al., 2012). Reported Mn hair concentrations levels were considerably larger in children (mean (range) = 4.4 (1.2–12.4) μg/g) compared to adults (median (range) = 0.44 (0.1–7.4) μg/g) (Rugless et al., 2014; Standridge et al., 2008).

### 3.5. Study evaluation

**Fig. 1.**
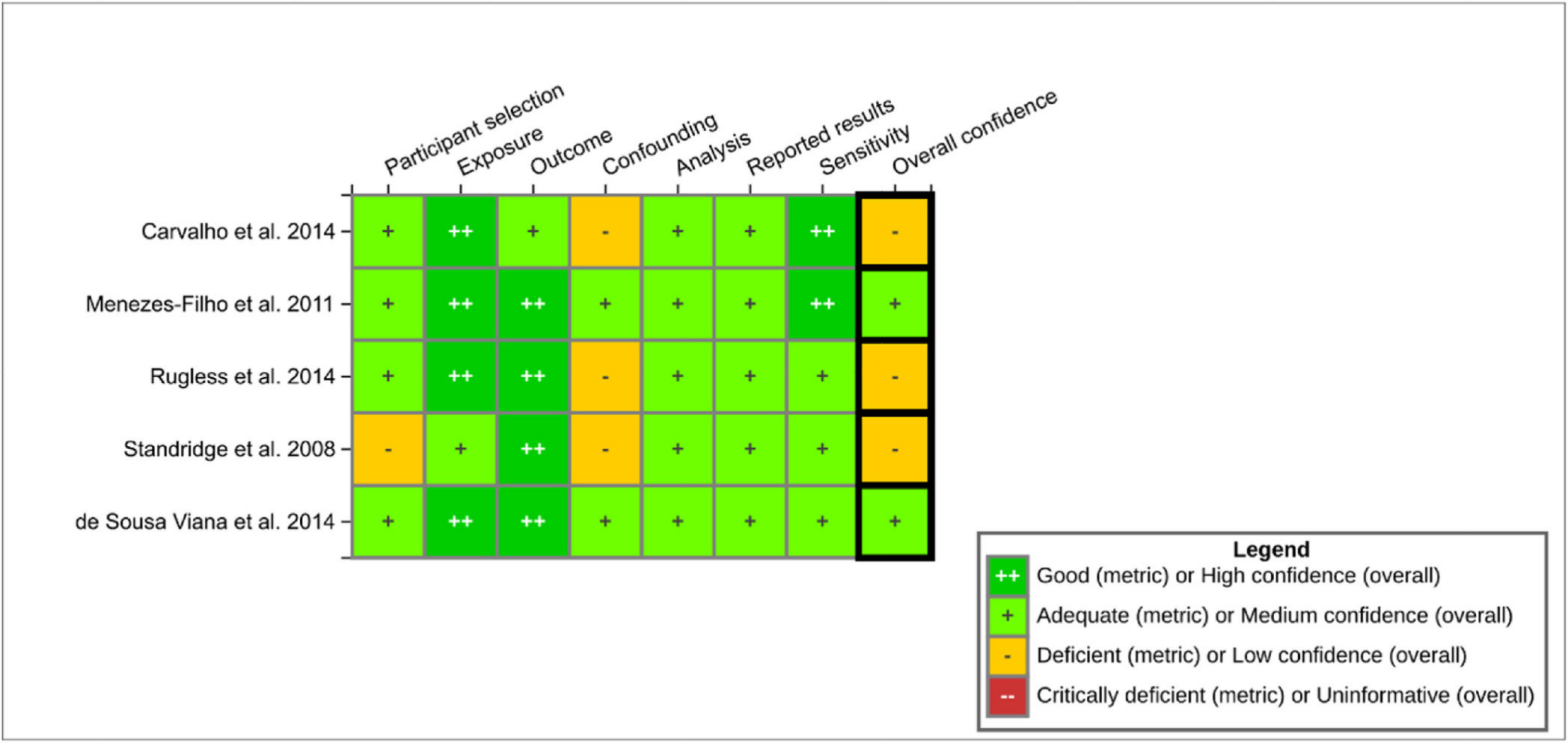
Heatmap of study evaluation results. Interactive version available on HAWC page: https://hawc.epa.gov/assessment/100500249/.

Fig. 1 illustrates the results of the study evaluation (see Methods). The rationales that support the judgments are available on the EPA HAWC page: https://hawc.epa.gov/assessment/100500249/. For the overall study evaluation domain, two studies were considered *medium* confidence (de Sousa Viana et al., 2014; Menezes-Filho et al., 2011) and three studies were considered *low* confidence (Carvalho et al., 2014; Rugless et al., 2014; Standridge et al., 2008). The main methodological deficiencies identified in these *low* confidence studies involved confounding (e.g., inadequate approach or insufficient adjustment for key potential confounders, including sex, family functioning/home environment, and anemia status/iron levels for IQ studies (Carvalho et al., 2014) - and physical activity and anemia status/iron levels for motor outcomes (Rugless et al., 2014; Standridge et al., 2008)) and participant selection (e.g., concerns with potential selection bias due to limited information on participant recruitment procedures (Standridge et al., 2008)). We were unable to confidently assess the direction of anticipated bias in these scenarios because of limited data.

Most studies were adequate or good for other domains, with particular strengths in exposure assessment (e.g., standardized approaches to hair collection and Mn analysis considering the assumptions discussed in the *Introduction*) and outcome ascertainment (e.g., validated neuropsychological or motor tests, blinded to exposure status).

### 3.6. Comparative analysis

#### 3.6.1. IQ decrements

Table 3 describes results for the paired analyses of IQ and hair Mn in the Brazilian communities. Comparing the betas between children from Menezes-Filho et al. (2011) using “Full scale IQ” and in adults from de Sousa Viana et al. (2014) using “Estimated IQ” showed that the decrement was approximately 1 IQ point larger in kids compared to adults per each unit increase in log_10_-transformed hair Mn (Child β - Adult β: −1.02; 95% CI: −7.69, 5.65). The ratio of the IQ decrements was 1.21, suggesting that children had a greater magnitude of decrement in IQ than seen in adults. We have more confidence in this comparison, given that it was based on two studies rated *medium* confidence.

Comparing the betas for “Estimated IQ” between children from Carvalho et al. (2014) and in adults from de Sousa Viana et al. (2014) showed that the slope for children was associated with even larger IQ decrements (−4.91: 95% CI: −13.42, 3.59) per unit increase in log_10_-transformed hair Mn. The ratio of the IQ decrements was higher (2.03) than the other paired analysis. However, confidence in this comparison is lower than in the comparison above of the two *medium* confidence studies, due to the overall study evaluation rating of *low* assigned to the Carvalho et al. (2014) study (Fig. 1).

Overall, based on two sets of comparisons from these three studies, children appeared to be 1.21 to 2.03-fold more sensitive to IQ decrements associated with inhaled Mn exposure compared to adults.

#### 3.6.2. Motor impairments

Table 4 describes results for the paired analysis of motor outcomes related to inhaled Mn among communities in and around Ohio, United States. Both postural sway length and sway area were evaluated as outcomes under different testing scenarios (e.g., eyes open vs. eyes closed; standing on foam vs. not standing on foam). For four of the five test condition comparisons, the mean response in children was equivalent to or slightly stronger than the mean response in adults. For example, comparing the betas for the sway length tests with eyes open, the slope for children was slightly larger than for adults (Child β - Adult β: 0.03; 95% CI: −0.14, 0.21). The ratio of motor decrements in this comparison was 1.75, suggesting that children had a greater magnitude of effect than adults. In another example, when comparing the betas for the sway area test with eyes closed and participants standing on foam, the slope for children was also larger than for adults (Child β - Adult β: 0.05; 95% CI: −0.32, 0.42). The ratio of these motor decrements was 1.28, also suggesting that children had a greater magnitude of effect compared to adults. By contrast, when comparing the betas for the sway area tests with eyes closed and with participants not standing on foam, the slope for children was smaller than for adults (Child β - Adult β: −0.19; 95% CI: −0.55, 0.17). The ratio of motor decrements for this test was 0.37, suggesting that children had a smaller magnitude of effect compared to adults. Overall, the postural balance decrements associated with inhaled Mn exposure based on these two Ohio studies was mixed, with children exhibiting both less susceptibility (e.g., ratio of βs = 0.37) and more susceptibility (e.g., ratio of βs = 1.75) compared to adults depending on the endpoint and test condition. However, confidence in these comparisons is limited, due to the overall study rating of *low* assigned to both studies by Rugless et al. (2014) and Standridge et al. (2008) (Fig. 1).

## 4. Discussion

It is well documented that children are particularly vulnerable to toxicants, including neurotoxicants and can differ from adults in their responses (Grandjean and Landrigan, 2014; Rice and Barone, 2000). Children may be uniquely susceptible to the effects of Mn exposures, specifically, due to their elevated inhalation rate, unique dietary and activity patterns, higher intestinal absorption due to physiological changes and rapid growth, greater permeability of the nervous system, reduced biliary excretion, and rapidly developing nervous system (Aschner and Aschner, 2005; ATSDR, 2012; Bateson and Schwartz, 2007; Dorman et al., 2000; Ginsberg et al., 2005; Magby and Richardson, 2018; Neal and Guilarte, 2013; Winder et al., 2010).

The current Mn database is not sufficient to develop a child-specific risk estimate from inhaled manganese. Based on a limited dataset, our analysis of comparative susceptibility to neurological outcomes from inhaled Mn suggests that children may be 0.37 to 2.03 times as susceptible as adults. These observations are supported by limited animal data (ATSDR, 2012). The range of our results is in line with what has been observed previously in comparisons of child and adult susceptibility (Barton et al., 2005; U. S. Environmental Protection Agency (EPA) 2005).

### 4.1. Inter-individual variability

Multiple sources of inter-individual variability – including lifestage, genetics, race/ethnicity, gender, health status, and lifestyle should also be considered when evaluating differential inter-individual susceptibility. Therefore, our findings of potential 0.37 to 2.03-fold differences in susceptibility between children and adults are important to consider but should not be construed as characterizing all of the sources of variation. In fact, there are numerous additional sources of inter-individual variation that are beyond the scope of our analysis and thus have not been accounted for here, including lifestyle factors such as diet. For example, iron deficiency is a common nutritional deficiency across the globe (Centers for Disease Control and Prevention (CDC) 2002) that contributes to increased Mn accumulation via a shared ion transporter (ATSDR, 2012; DeWitt et al., 2013; Erikson et al., 2005; Thompson et al., 2007). Individuals with iron deficiency could be more susceptible to the effects of Mn than the general population, and therefore one would need to account for this variation as well.

Genetic and epigenetic factors represent another potentially important source of intraindividual variation in susceptibility to Mn (Curran et al., 2009; Lucchini et al., 2018). Several studies have documented the role of genetic polymorphisms in mediating Mn regulation, transport, and toxicity (Claus Henn et al., 2011; Haynes et al., 2010; Rentschler et al., 2012; Wahlberg et al. 2016, 2018). Two of these studies evaluated the role of genetic polymorphisms as related to Mn toxicity among populations likely to experience substantial Mn inhalation exposure from point sources (Rentschler et al., 2012; Wahlberg et al., 2018). While we were unable to incorporate these studies into our paired analysis due to use of blood rather than hair as the biomarker, these data suggest the presence of effect modification by genotype, with up to 4-fold and 10-fold differences in coefficients for motor outcomes among the elderly and cognitive outcomes among children, respectively (Rentschler et al., 2012; Wahlberg et al., 2018). The substantial impact of these genetic variations in susceptibility to Mn neurotoxicity also warrants consideration.

We report that children may be 0.37 to 2.03 times as susceptible as adults to the neurotoxic effects of manganese, thereby providing a quantitative estimate for some aspects of lifestage variation. The comparison between the two studies with the least uncertainty (i.e., the *medium* confidence studies) was in the middle of this distribution (at 1.21 times susceptibility), and it did not appear that our judgements about individual study quality impacted the quantitative estimates. However, considering all the inter-individual variability factors noted above that are not considered here, the extent of total inter-individual variability remains unclear.

### 4.2. Limitations and strengths

Our analysis – one of the first of its kind using existing epidemiological data to compare lifestage variations – has some limitations. First, we selected studies with clear point source exposures to airborne Mn to minimize noise in our analysis, but it is possible that through this approach we excluded other child/adult paired sets. This issue was recognized as a potential concern but reducing noise was considered a more significant priority. In addition, the studies that we reviewed all had relatively small sample sizes which could impact their statistical power and, consequently, the precision of our comparisons of the studies’ results.

While we attempted to select populations from the same geographic area with the same point-source exposures to Mn, several aspects may limit comparability between groups. For example, there were large differences in the central tendency value for hair Mn between the Ohio child and adult populations. Potential reasons for these differences may be due to downward trends in exposure over time (adults were evaluated in 2006 and children were evaluated in 2009–2010), laboratory differences (distinct measurement methods and labs were used for the child vs. adult populations), differences in behavior patterns, or biological/metabolic differences (Personal communication with Dr. Erin Haynes, 2021). Our analyses relied upon the use of the available characterization of exposure-response functions, which were modeled by each investigator as linear functions of log-transformed Mn concentrations in hair (linear in log-space, but non-linear in normal space). Under these assumptions, a comparison of slopes would still be appropriate despite the differences in exposure level. If the underlying shape of the exposure-response functions for Mn in hair differed between adults and children due to differences in hair Mn concentrations, then this would introduce uncertainty into our analysis.

A second comparability concern is related to validity of the outcome comparisons at different timepoints across the lifestage. For example, due to continued skeletal maturation, child postural control is less well-developed than adults and expected to improve with age (Barozzi et al., 2014). If the observed measures of postural stability in children do not reflect their ultimate postural abilities, comparing these outcomes across age-groups could add uncertainty to our analysis.

Thirdly, these studies incorporated different combinations of covariates in their models, and the degree that an individual study may have more or less residual confounding may complicate the comparison across lifestages. Comparing the relative effects by the overall confidence in each study pair, the highest confidence pair were both judged to have addressed potential confounding adequately and to be *medium* confidence overall (children from Menezes-Filho et al., 2011 and in adults from de Sousa Viana et al., 2014) and that relative difference was 1.21 and in the middle of the distribution of relative effects. While differences in potential residual confounding are an uncertainty, the individual study confidence judgements did not appear to be related to the quantitative estimates of relative effect sizes between children and adults.

In the absence of extensive air monitoring information, hair biomarkers appear to be most relevant to characterizing chronic exposures to Mn. However, some uncertainty remains as to whether they can fully represent key critical windows for the outcomes of interest in our analysis. Based on the average growth rate for hair (1 cm per month) (Tobin, 2005) and the lengths utilized in the reviewed studies when documented (1–12 cm), these hair samples realistically reflect average exposure over a period of *<*12 months. This period of *<*12 months would represent a more substantial period of a young child’s life than an adult’s life; correspondingly, hair biomarkers may be more appropriate for studies of outcomes in children compared to adults. However, because we have assumed that 1) these populations have lived in the same geographic area for their entire lives, and 2) airborne Mn emissions have been constant over their life-course, our assessment of months-long exposure from hair samples is understood to represent chronic exposures for both children and adults-including earlier windows that may be critical for neurodevelopmental outcomes. These assumptions may not hold, which introduces uncertainty into our analyses. For example, the studies had differing inclusion criteria with regard to residential history, ranging from at least one year of residence in the location of the study (Menezes-Filho et al., 2009) to having lived in the same location throughout life (Rugless et al., 2014). Additionally, with regard to the assumption about constant exposure, the ranges of air Mn obtained through seven days of sampling in Brazil (0.001–0.439 μg/m^3^) and annual modeling in Ohio (0.006–0.160 μg/m^3^) were relatively large (Fulk et al., 2017; Menezes-Filho et al., 2009). Since hair biomarkers are anticipated to provide estimates of average exposures over the prior months, any extreme variation outside of these windows that are not reflected (or exposures from residence outside of the study area) may result in decreased study sensitivity and attenuation of the underlying effect estimates due to measurement error. We do not consider this to be a large source of uncertainty, however, as we anticipate that any measurement error would be comparable across the paired studies from the same study base. Another issue is the degree to which hair represents internalized Mn dose, particularly at the target organ, which may differ by lifestage. Yet, we note that the greatest changes in physiology occur in infants/toddlers and therefore hair biomarkers are likely appropriate and representative for the age groups in our analysis.

Additional concerns associated with the use of hair Mn biomarkers in the original studies relate to sources of sample variation and contamination. These and other sources of measurement error could decrease sensitivity in the original studies and also in our comparisons. For example, there can be variability in analytic procedures, such as hair washing protocols used to remove surface Mn contamination (Eastman et al., 2013), limiting consistency and comparability between studies conducted in different labs. Natural hair color and diet may also influence Mn levels (Kempson and Lombi, 2011; Lydén et al., 1984; Sturaro et al., 1994), yet few studies adjust for these factors in their analyses. Finally, there is potential for external contamination of hair samples, such as from Mn-contaminated water used in washing procedures prior to analysis or from hair dyes used for cosmetic reasons (Coetzee et al., 2016; Eastman et al., 2013).

Notwithstanding the limitations and simplifying assumptions that were required for this work, our study has several strengths. Importantly, to our knowledge, this is the first comparison of child and adult susceptibility to the neurotoxic effects of inhaled Mn. Differential lifestage susceptibility is an important consideration, but few studies have used available data to evaluate observed differences. Our results provide comparisons that can inform and contextualize confidence in understanding lifestage variabilities.

A major strength of this work is that it highlights several significant data gaps and crucial research needs that must be addressed to advance our understanding of Mn toxicity and factors that impact susceptibility. Only a small number of studies met the strict eligibility criteria for our comparative analysis focused on inhalation exposure. While our initial literature search yielded 129 publications that met PECO criteria, only five studies utilizing hair biomarkers could be paired. Given this limited set of studies (and the small sample size in each of the individual studies), we urge caution in interpreting our results. We strongly encourage future research in both children and adults exposed to the same point sources of airborne Mn in order to delineate lifestage differences and better inform our understanding of potential differential susceptibility stemming from other factors, such as genetic polymorphisms. Another important research direction is to better understand the shape of the dose-response curve in children and adults. The studies that we paired considered only a linear dose-response of log-transformed Mn concentrations in their analyses (Carvalho et al. 2014, de Sousa Viana et al., 2014; Menezes-Filho et al., 2011; Rugless et al., 2014; Standridge et al., 2008). However, nonlinear dose-response curves for Mn have been observed previously (Haynes et al., 2015; Vollet et al., 2016). Nonlinearity is fairly common for minerals that are both nutrients and toxicants. Thus, this topic should be a priority for future work.

Another important research direction identified through this effort is to better understand the critical windows of vulnerability for Mn and neurotoxicity. The placenta regulates Mn transport during the fetal period, since it is an essential nutrient involved in key developmental processes. Prior studies present conflicting evidence on the relative importance of prenatal versus postnatal exposures in Mn-related adverse effects (Bauer et al., 2017; Claus Henn et al., 2018; Gunier et al., 2015; Mora et al., 2015). The studies that we reviewed could not disentangle effects of prenatal and postnatal Mn exposures, but most individuals in the study populations – particularly the children – are expected to have also lived in the same geographic area during the fetal period. More research in this area is needed to understand critical windows of vulnerability and the contribution of specific exposure routes such as transplacental compared to breast milk and other early life influences. Similarly, further work to better understand potential distinctions in later life vulnerability would be informative. The studies of adults reviewed here encompass fairly large age ranges (e.g., 15–55 years; 31–68 years) (likely due to small sample sizes), and it is possible there are distinct impacts on middle versus older aged adults.

More research is also needed to address our incomplete understanding of the relationship between inhaled Mn and hair Mn concentrations. As noted before, our analysis was necessary because existing PBPK models do not include a hair compartment, and a general means for relating hair and blood Mn has not been published. Advancements contributing to the inclusion of a hair compartment in PBPK models, or more empirical data on the relationship between hair and blood Mn, could allow the numerous studies of neurological outcomes in children using hair biomarkers to be utilized directly to develop inhalation risk estimates. There should also be further work to understand how inhaled Mn relates to hair Mn in infants and toddlers that exhibit faster growth rates and different physiology than children and adults. Such studies might impact the degree to which hair biomarkers can be used to represent chronic exposure across lifestage. Additionally, given the growing use of tooth biomarkers in assessing early-life exposures to metals (Claus Henn et al., 2018; Horton et al., 2018), efforts to include a tooth compartment in Mn PBPK models could also be an important advancement. In the absence of PBPK model advancements allowing the integration of hair (or teeth) and air Mn, there is a need for additional studies in children based on airborne Mn measures, which could contribute to developing child-specific inhalation risk estimates with more direct risk management implications.

## 5. Conclusion

We evaluated a small database of paired studies to compare child and adult susceptibility to the neurotoxic effects of inhaled Mn. Based on our analysis incorporating a set of simplifying assumptions, we report that children may be 0.37 to 2.03 times as susceptible as adults, thereby providing a quantitative estimate for some aspects of lifestage variation.

However, as noted earlier, our evaluation does not encompass all aspects of human variability. Consideration for differences in susceptibility due to numerous factors including genetics, race/ethnicity, gender, health status, and lifestyle is essential. Our analysis only evaluated one aspect of variability (i.e., lifestage), and there is some uncertainty in our results due to the assumptions included. Therefore, at this time, there is insufficient information to fully characterize individual variability for this exposure and outcome. Further work should be conducted to elucidate the diversity of factors that can lead to differences in susceptibility and provide data when considering the effects of inhaled Mn.

## Supplementary Material

supp material

## Figures and Tables

**Table 1:** Population Exposures Comparators and Outcomes (PECO) criteria.

P	Epidemiology studies in humans at any lifestage. The following study designs were considered most informative: controlled exposure, cohort, case-control, or cross-sectional studies. Case series or case reports were not considered PECO relevant but were tagged as supplemental material. Animal and *in vitro* studies were not considered PECO-relevant and were tracked as supplemental information.
E	Studies with quantitative exposure data relevant to chronic inhalation exposures from point-sources, based on measured and modeled air concentrations, as well as workplace histories or proximity to industrial site releases, were evaluated. Biomarker data of any kind were also considered.
C	A comparison or reference population exposed to lower airborne levels (no exposure or exposure below manganese detection limits) or exposed to airborne manganese for shorter time periods.
O	Neurological and musculoskeletal outcomes.

**Table 2:** Key population characteristics for studies in the Mn comparative analysis.

Outcome	IQ			Postural Balance	
Location	Bahia, Brazil			Ohio, USA	
Source of inhaled Mn	Local ferromanganese smelter				
Study	Carvalho et al. (2014)	Menezes-Filho et al. (2011)	de Sousa Viana et al. (2014)	Rugless et al. (2014)	Standridge et al. (2008)
Study dates	2011	2007	2011	2009–2010	2006
Lifestage (Age range)	Child (7–12 yrs)	Child (6–11 yrs)	Adult (15–55 yrs)	Child (7–9 yrs)	Adult (31–68 yrs)
Sample size	n = 70	n = 83	n = 81	n = 48	n = 22
Median (range) measured or modeled ambient Mn concentrations	0.114 (0.011–0.439) μg/m^3^			0.008 (0.001–0.054) μg/m^3^	
Outcome assessment tool	Wechsler Intelligence Scale for Children (WISC)	Wechsler Intelligence Scale for Children (WISC)	Wechsler Adult Intelligence Scale (WAIS)	Hall-Effect type portable force platform system	Hall-Effect type portable force platform system
Hair Mn concentrations	median (range) = 11.48 (0.52–55.74) μg/g mean (SD) = 14.6 (11.8) μg/g	mean (range) = 5.83 (0.1–86.68) μg/g SD = 11.5 μg/g	median (range) = 8.95 (0.6–44.6) μg/g	median (range) = 0.44 (0.1–7.4) μg/g mean (SEM) = 0.77 (0.16) μg/g	mean (range) = 4.4 (1.2–12.4) μg/g SD = 3.3 μg/g

**Table 3:** Paired studies utilized in the comparative analysis of IQ.

Outcome comparison^a^	Lifestage (age range)	Study (sample size)	Overall Study Confidence	β^b^ (95% CI)	Child β - Adult β	T statistic and *P*-value for the difference in β	Ratio of child β: adult β (% difference)
Full scale IQ vs. Estimated IQ	Child (6–11 yrs)	Menezes-Filho et al. (2011) (n = 83)	*medium*	−5.78 (−10.71, −0.21)	−1.02 (95% CI: −7.69, 5.65)	*t* = 0.3 *p* = 0.76	1.21 (+21%)
	Adult (15–55 yrs)	de Sousa Viana et al. (2014) (n = 81)		−4.76 (−9.17, −0.36)			
Estimated IQ vs. Estimated IQ	Child (7–12 yrs)	Carvalho et al. (2014) (n = 70)	*low*	−9.67 (−16.97, −2.37)	−4.91 (95% CI: −13.42, 3.59)	*t* = 1.14 *p* = 0.26	2.03 (+103%)
	Adult (15–55 yrs)	de Sousa Viana et al. (2014) (n = 81)	*medium*	−4.76 (−9.17, −0.36)			

a Full Scale IQ obtained from all domains of the Wechsler Intelligence Scale. Estimated IQ obtained from Vocabulary and Block Design subtests of Wechsler Intelligence Scale.

b β expresses a 1-unit change in the outcome per 1 unit change in log_10_ Mn in hair (μg/g).

**Table 4:** Paired studies utilized in the comparative analysis of postural sway.

Outcome	Scenario:		Life-stage	Study (sample size)	Overall Study Confidence	β (SE β)^a^	Child β -Adult β	T statistic and *P*-value for the difference in β	Ratio of child β: adult β (% difference)
	Eyes	Foam							
Sway Length	Open	Yes	Child (7–9 yrs)	Rugless et al. (2014) (n = 48)	*Low*	0.07 (0.03)	0.03 (95% CI: −0.14, 0.21)	*t* = 0.35 *p* = 0.73	1.75 (+75%)
			Adult (31–68 yrs)	Standridge et al. (2008) (n = 22)		0.04 (0.08)			
	Closed		Child (7–9 yrs)	Rugless et al. (2014) (n = 48)		0.08 (0.03)	0 (95% CI: −0.26, 0.26)	*t* = 0 *p* = >0.99	1 (0%)
			Adult (31–68 yrs)	Standridge et al. (2008) (n = 22)		0.08 (0.12)			
Sway Area	Open	Yes	Child (7–9 yrs)	Rugless et al. (2014) (n = 48)		0.14 (0.06)	0.01 (95% CI: −0.23, 0.25	*t* = 0.09 *p* = 0.93	1.07 (+7%)
			Adult (31–68 yrs)	Standridge et al. (2008) (n = 22)		0.18 (0.17)			
	Closed		Child (7–9 yrs)	Rugless et al. (2014) (n = 48)		0.23 (0.06)	0.05 (95% CI: −0.32, 0.42)	*t* = 0.28 *p* = 0.78	1.28 (+28%)
			Adult (31–68 yrs)	Standridge et al. (2008) (n = 22)		0.18 (0.17)			
		No	Child (7–9 yrs)	Rugless et al. (2014) (n = 48)		0.11 (0.08)	−0.19 (95% CI: −0.55, 0.17)	*t* = −1.19 *p* = 0.30	0.37 (−63%)
			Adult (31–68 yrs)	Standridge et al. (2008) (n = 22)		0.30 (0.16)			

a β expresses the change in ln(outcome) for each 1 unit increase in ln hair Mn (μg/g).

## Data Availability

The data used in this research was extracted from the cited publications
